# Characteristics of radiofrequency lesions in patients with symptomatic periesophageal vagal nerve injury after pulmonary vein isolation

**DOI:** 10.1002/joa3.13036

**Published:** 2024-04-05

**Authors:** Shingo Yoshimura, Yutaka Take, Kenichi Kaseno, Koji Goto, Yuji Matsuo, Hideyuki Aoki, Takehito Sasaki, Yuko Miki, Kohki Nakamura, Shigeto Naito

**Affiliations:** ^1^ Division of Cardiology Gunma Prefectural Cardiovascular Center Maebashi Japan

**Keywords:** atrial fibrillation, catheter ablation, periesophageal vagal nerve injury, pulmonary vein isolation, radiofrequency lesions

## Abstract

**Background:**

Periesophageal vagal nerve injury (PNI) is an unpredictable and serious complication of atrial fibrillation (AF) ablation. We aimed to identify the factors associated with symptomatic PNI.

**Methods:**

This study included 1391 patients who underwent ablation index‐guided pulmonary vein isolation (PVI) using the CARTO system. The target ablation index was set at 550, except for the left atrial (LA) posterior wall near the esophagus, where radiofrequency (RF) power and duration were limited. Ten patients (0.72%) were diagnosed with symptomatic PNI. We randomly selected 40 patients without PNI (1:4 ratio) matched based on age, sex, body mass index, LA diameter, type of AF, and esophageal location. We measured the shortest distance from the RF lesions to the esophagus (LED) and classified the RF lesions according to the LED into four groups: 0–5, 5–10, 10–15, and 15–20 mm. We conducted a comparative analysis of classified RF lesions between patients with PNI (*n* = 10) and those without (*n* = 40).

**Results:**

The contact force at LED 0–5 mm was significantly higher in patients with PNI than in those without (14.6 ± 1.7 vs. 12.0 ± 2.9 g; *p* = .01). Multivariate logistic analysis revealed that the independent factor for PNI was contact force at an LED of 0–5 mm (odds ratio: 1.506; 95% confidence interval: 1.053–2.153; *p* = .025).

**Conclusions:**

The symptomatic PNI was significantly associated with a higher contact force near the esophagus. Strategies for regulating contact force near the esophagus may aid in the prevention of PNI.

## Graphical abstract

Periesophageal vagal nerve injury (PNI) is an unpredictable complication of atrial fibrillation ablation. We measured the distance from radiofrequency lesions to the esophagus (LED) and assessed RF lesions in patients with symptomatic PNI. PNI was significantly associated with a higher contact force at an LED of 0–5 mm.

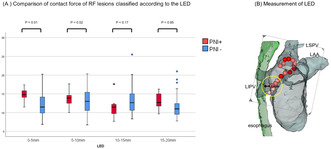

## 1 INTRODUCTION

Radiofrequency (RF) catheter ablation is an effective curative treatment for patients with atrial fibrillation (AF). Catheter ablation guided by contact force and three‐dimensional (3D) electroanatomical mapping systems reduces procedural complications.^1^, ^2^ However, predicting the collateral damage of RF energy to the surrounding structures, particularly the esophagus, remains challenging. Esophageal injuries caused by collateral damage due to RF energy include erosion, ulceration, atrial esophageal fistula, and periesophageal vagal nerve injury (PNI). Despite esophageal temperature monitoring to reduce thermal injury,^3^, ^4^ PNI can occur as a serious and unpredictable complication of AF ablation. The incidence of PNI diagnosed using gastrointestinal endoscopy after AF ablation is not uncommon, ranging from 15% to 23%.^5^, ^6^ Esophageal injury may be related to the proximity of the esophagus to the left atrial (LA) posterior wall^7^, ^8^; however, the association between PNI and the characteristics of RF lesions and their proximity to the esophagus remains unclear.

This study aimed to investigate whether PNI is related to the characteristics of RF lesions and the distance between RF lesions and the esophagus.

## 2 METHODS

### 2.1 Study population

We screened 1678 patients who underwent ablation index‐guided pulmonary vein isolation (PVI) using a CARTO system (Biosense Webster Inc., Diamond Bar, CA, USA) for paroxysmal or persistent AF between April 2018 and September 2020. We excluded 230 patients who had previously undergone AF ablation procedures and 57 who had undergone additional LA ablation, including posterior wall isolation, roof‐line ablation, mitral annular flutter ablation, and defragmentation.

Symptomatic PNI was defined by the following symptoms and findings within 1 week after AF ablation: symptoms of gastroparesis, such as nausea, vomiting, postprandial fullness, epigastric pain, bloating, or early satiety, and findings of gastric hypomotility identified by abdominal radiography, computed tomography (CT), or gastrointestinal endoscopy.^4^, ^9^, ^10^

**FIGURE 1:**
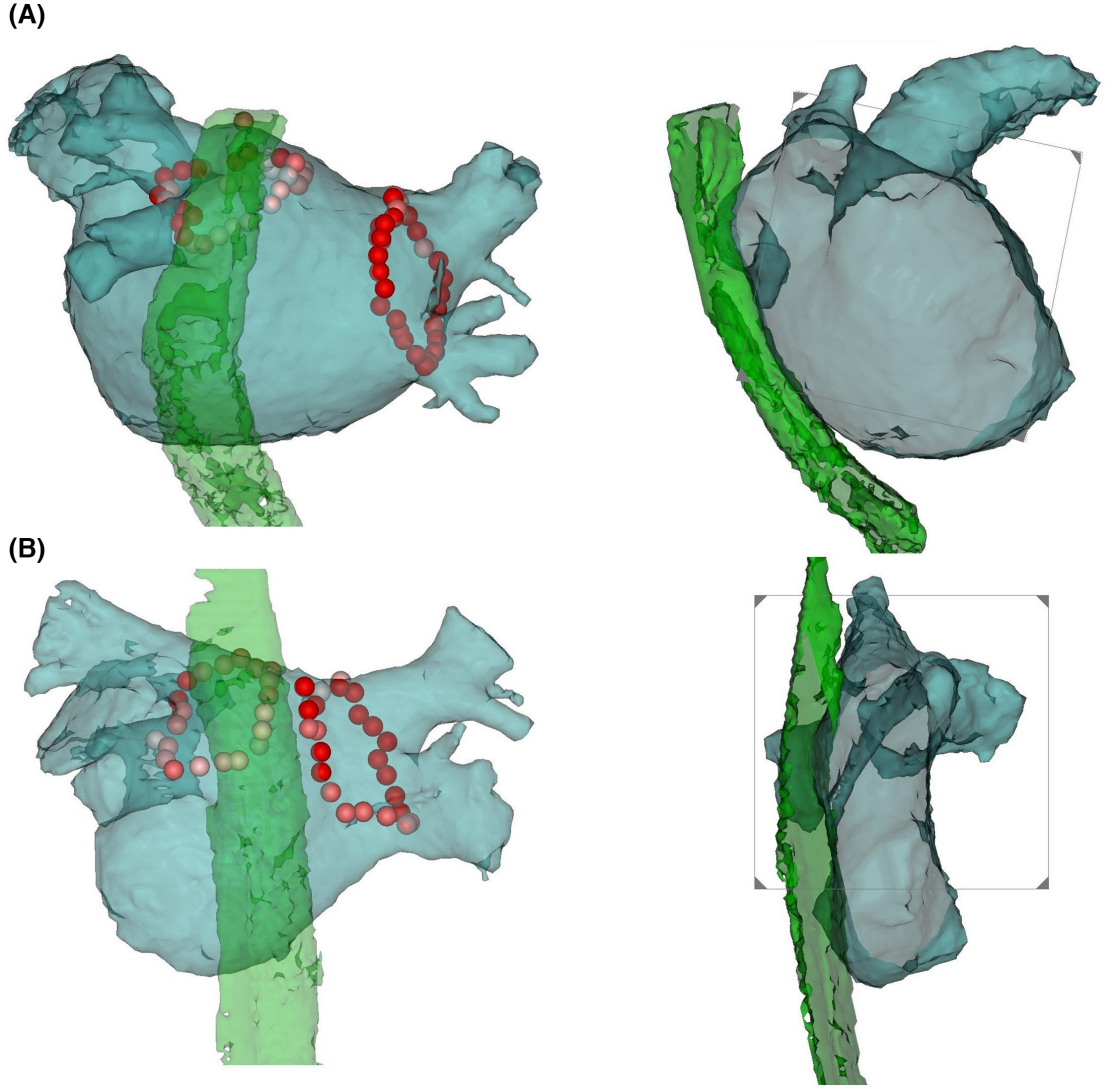
The location of the esophagus and circumferential ablation line in two patients with periesophageal vagal nerve injury (A: case 1; B: case 2). The left panel shows a posteroanterior view of the left atrium, and the right panel shows an inner view of the left lateral position. The esophagi are closer to the left atrial posterior wall at the level of the left inferior pulmonary vein.

Of the 1391 patients, 10 (0.72%) were diagnosed with symptomatic PNI. Representative cases are shown in Figure 1. Forty patients without PNI were randomly selected (1:4 ratio). These patients were matched to those with PNI based on age, sex, body mass index (BMI), LA diameter, AF type (paroxysmal or persistent), and esophageal location at the LA posterior wall. We compared patients with PNI to those without PNI.

### 2.2 Ablation procedure

The procedural protocol has been described in a previously documented report.^11^ Before the ablation procedure, comprehensive assessments of the LA, pulmonary vein (PV), and esophageal anatomy were conducted using contrast‐enhanced CT. The esophageal location on the CT images was classified into the middle, left, and right sides of the LA posterior wall. After deep propofol sedation, a temperature probe (SensiTherm; Abbott, St. Paul, MN, USA) was positioned within the esophagus at the level of the LA through the oral or nasal cavity to monitor the esophageal temperature continuously. A transseptal puncture was executed, and LA electroanatomical maps were integrated with the CT images using landmarks and surface registration. We performed circumferential PVI employing the double‐lasso technique with a 3.5 mm irrigation tip catheter (Thermocool Smart Touch SF; Biosense Webster Inc.). PVI was performed utilizing the ablation index and Visitag™, which automatically displayed RF lesions. The settings for catheter stability were predefined as follows: maximum range, 3 mm; minimum time, 6 s; minimum contact force, 5 g; and force‐over time, 25%. We applied RF energy at a power range of 35–40 W, and the target value of the ablation index was set to 550, except for the LA posterior wall near the esophagus, where we restricted the power setting to <30 W and the RF duration to within 30 s, regardless of the ablation index. When the esophageal temperature reached >41°C, RF delivery was promptly halted. Following PVI, PV reconnection was assessed using adenosine administration, and cavotricuspid isthmus ablation was performed. If PV reconnection was detected, additional RF applications were delivered to complete the PVI. After the procedure, proton pump inhibitors were administered to all patients for 1–3 months to prevent esophageal injury.

### 2.3 Measurement of distance from radiofrequency lesions to the esophagus and analysis of radiofrequency lesions

**FIGURE 2:**
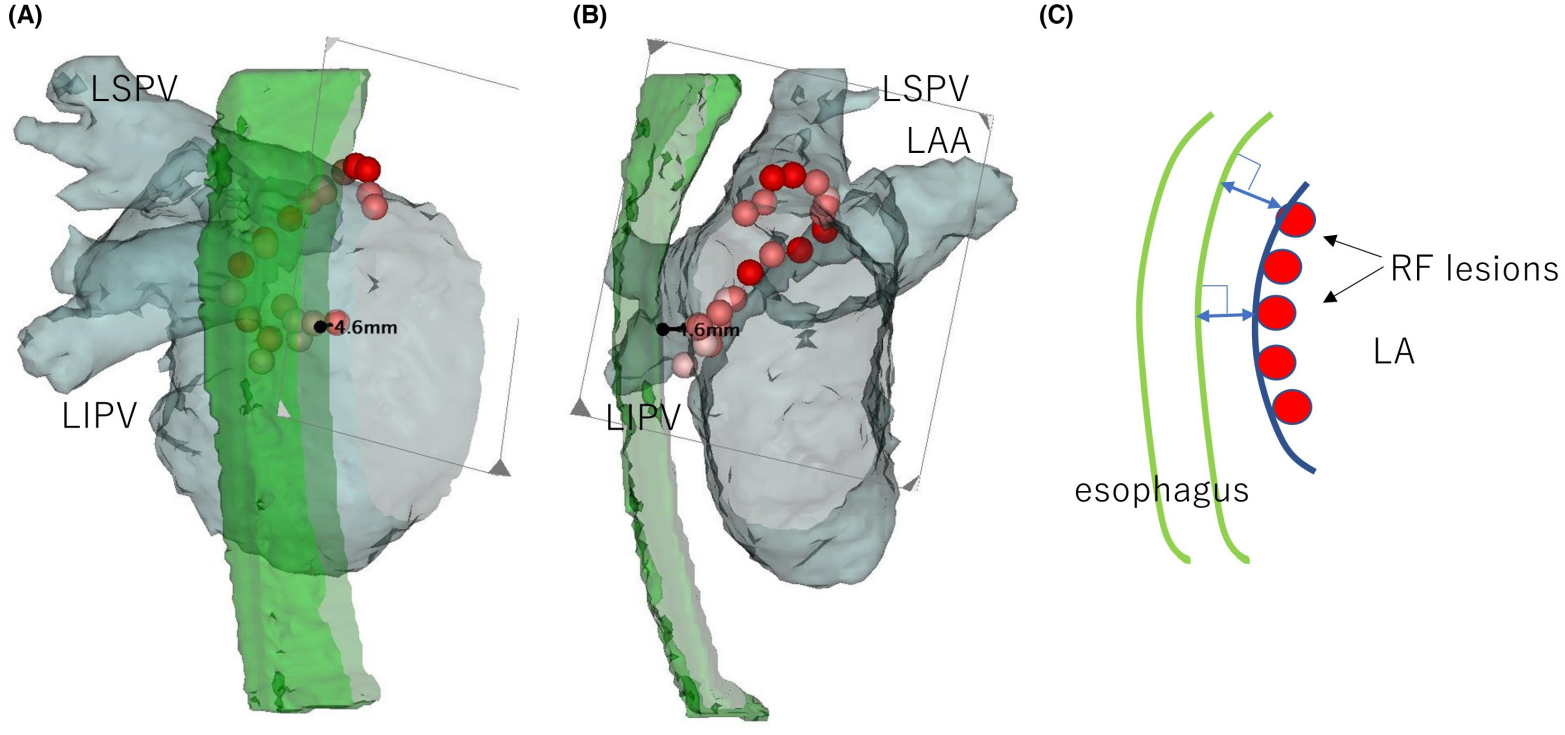
Measurement of lesion–esophageal distance (LED) using the CARTO3 system. Examples of LED measurements in patients without periesophageal vagal nerve injury. (A) Posteroanterior view of the LA. (B) Inner view of the left lateral position. The circle marked with 4.6 mm indicates the shortest distance from the tag of the RF lesion at the left atrial posterior wall to the anterior aspect of the esophagus. (C) The blue double‐headed arrow shows the distance between the anterior aspect of the esophagus and the left atrial posterior wall. LA, left atrium; LAA, left atrial appendage; LIPV, left inferior pulmonary vein; LSPV, left superior pulmonary vein; RF, radiofrequency.

We measured the distance between the RF lesion sites and the esophagus by manually selecting points using the CARTO3 system. The lesion–esophageal distance (LED) was defined as the shortest perpendicular distance from the RF‐lesion tag at the circumferential ablation line to the anterior aspect of the esophagus (Figure 2). Additionally, LED = 0 mm was defined as RF lesions on the LA posterior wall attached completely to the anterior aspect of the esophagus. RF lesions were classified according to the LED as follows: 0–5, 5–10, 10–15, and 15–20 mm. We compared the characteristics of classified RF lesions between patients with (*n* = 10) and without (*n* = 40) PNI. The characteristics of the classified RF lesions included RF duration, interlesion distance, contact force, ablation index value, RF power setting, and the number of RF lesions in the region.

### 2.4 Statistical analysis

Propensity scores were calculated for all 1391 patients using a multivariable logistic regression model, and propensity score matching was performed to select a control group without PNI using the 1:4 nearest‐neighbor matching algorithm with a caliper width of 0.02.

Statistical analyses are conducted by presenting data as the mean ± standard deviation for normally distributed variables. Continuous variables underwent comparison using the Student's *t*‐test or the Mann–Whitney *U* test, based on suitability. Categorical variables were presented as the patient number and percentage and compared using the χ^2^ test. The ability of the contact force in the RF lesion at LED 0–5 mm to predict the PNI was assessed using receiver operating characteristic (ROC) curve analysis, and the optimal threshold was determined as the value that maximized the sum of sensitivity and specificity. Logistic regression analysis was performed to identify the factors associated with PNI. Variables displaying a *p*‐value < .1 in the univariate analysis were included in the multivariate analysis. Statistical significance was set at *p* < .05. All statistical analyses were conducted using SPSS Statistics software (version 25.0; IBM Corp., Armonk, NY, USA).

## 3 RESULTS

### 3.1 Baseline clinical characteristics of patients with and without periesophageal vagal nerve injury

Among patients with PNI, the mean age was 68.6 ± 13.8 years, and two patients (20%) had persistent AF. The mean LA diameter on echocardiography was 36.3 ± 6.7 mm. Notably, the esophagus was located on the left side of the LA posterior wall in all patients with PNI. There were no significant differences in age, sex, AF type, LA diameter, or esophageal location between patients with PNI (*n* = 10) and without PNI (*n* = 1381) in the entire population (Table S1). Additionally, as shown in Table 1, patients with PNI (*n* = 10) had no significant differences in baseline characteristics compared to selected patients without PNI (*n* = 40).

**TABLE 1:** Comparison of demographic and baseline characteristics between patients with and without periesophageal vagal nerve injury.

	PNI (*n* = 10)	Control (*n* = 40)	*p* value
Age, years	68.6 ± 13.8	65.8 ± 9.7	.47
Men, *n* (%)	8 (80%)	31 (77%)	.86
Body mass index, kg/m^2^	19.1 ± 2.5	19.9 ± 3.1	.44
Persistent AF, *n* (%)	2 (20%)	14 (35%)	.37
Left atrial diameter, mm	36.3 ± 6.7	38.5 ± 8.3	.45
Esophageal location at the left side of the left atrium	10 (100%)	40 (100%)	
Left common pulmonary vein, *n* (%)	1 (10%)	3 (7.5%)	.79
Hypertension, *n* (%)	5 (50%)	22 (55%)	.78
Diabetes mellitus, *n* (%)	2 (20%)	9 (23%)	.86
CHA_2_DS_2_‐VASc score	2.8 ± 2.1	2.2 ± 1.4	.28
Structural heart disease, *n* (%)	0 (0%)	2 (5%)	.48
History of heart failure, *n* (%)	1 (10%)	7 (17%)	.57

*Note*: Values are presented as *n* (%), mean ± standard deviation.

Abbreviations: AF, atrial fibrillation; PNI, periesophageal vagal nerve injury.

### 3.2 Characteristics of radiofrequency lesions

**FIGURE 3:**
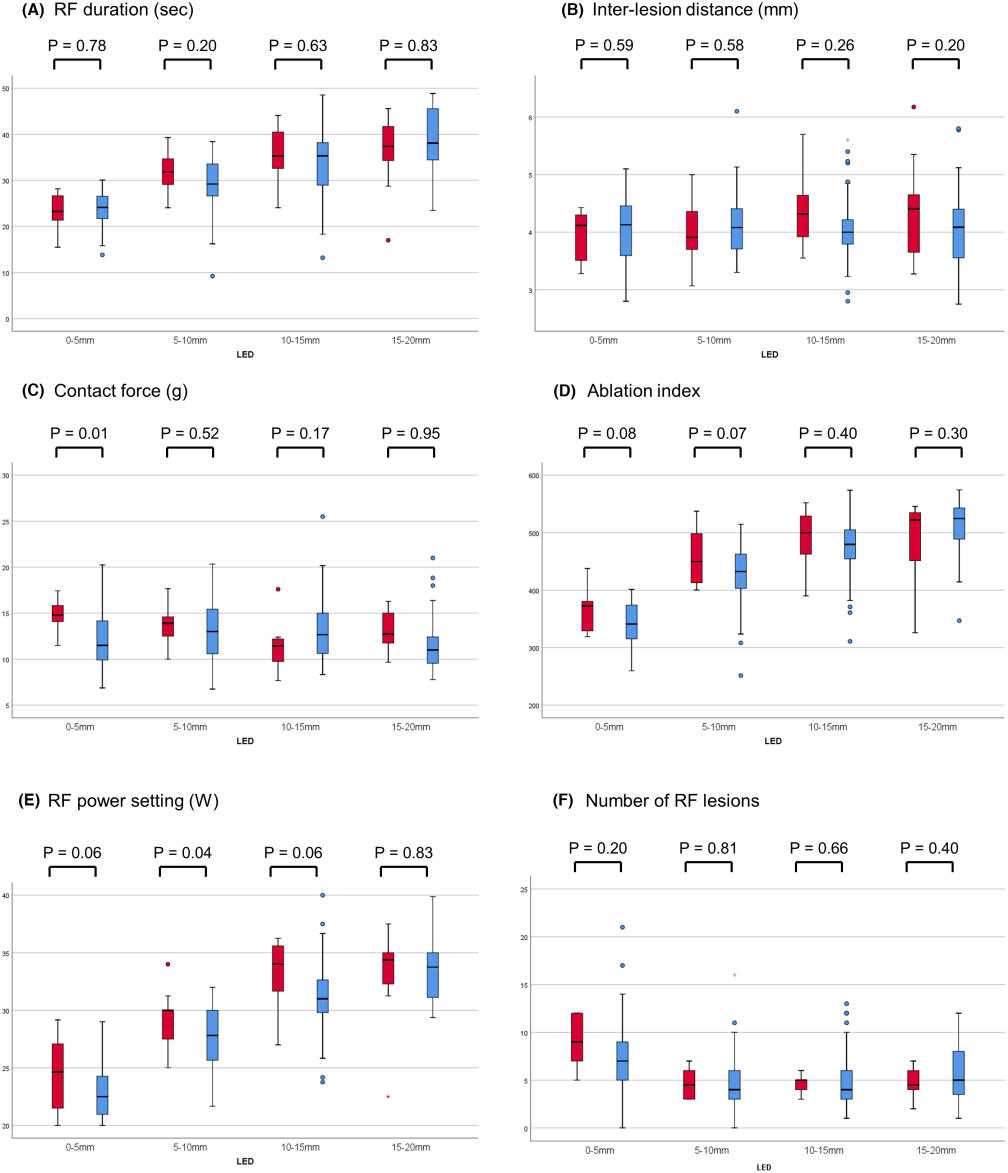
Box and whisker charts comparing RF lesions classified according to the LED between patients with and without PNI (left red and right blue bars, respectively) in (A) RF duration, (B) inter‐lesion distance, (C) contact force, (D) ablation index, (E) the mean RF power setting, and (F) the number of RF lesions at the region. Within each chart, the thick line in the middle represents the median, while the upper and lower box lines indicate the first and third quartiles. The whiskers extend to display the maximum and minimum values, excluding outliers (circles) and extremes (asterisks). LED, lesion–esophageal distance; PNI, periesophageal vagal nerve injury; RF, Radiofrequency.

Table 2 shows the clinical background and characteristics of RF lesions at LED 0–5 and 5–10 mm in all patients with PNI. The shortest LED was <5 mm in all patients with PNI, and there was no significant difference in the shortest LED compared to the patients without PNI (0.27 ± 0.81 vs. 0.77 ± 1.41 mm; *p* = .29). Figure 3 shows the characteristics of the RF lesions classified by LED. The RF duration, interlesion distance, ablation index, and number of RF lesions did not differ between patients with and without PNI, regardless of the LED (Figure 3A,B,D,F). The contact force at LED 0–5 mm was significantly higher in patients with PNI than those without PNI (14.6 ± 1.7 vs. 12.0 ± 2.9 g; *p* = .01) (Figure 3C). The mean RF power setting at LED 5–10 mm was higher in patients with PNI than those without PNI (29.4 ± 2.3 vs. 27.5 ± 2.7; *p* = .04) (Figure 3E).

**TABLE 2:** Clinical background and characteristics of radiofrequency lesions of patients with symptomatic periesophageal vagal nerve injury.

Patient number	Age	Gender	LAD (mm)	Type of AF	Shortest LED (mm)	LED 0–5 mm			LED 5–10 mm		
						Mean contact force (g)	Mean ablation index	Mean RF power (W)	Mean contact force (g)	Mean ablation index	Mean RF power (W)
1	78	F	48	PerAF	0	15	326	20.8	16.6	491	30
2	34	M	27	PAF	0	17.4	330	24.2	13.4	454	30
3	69	M	47	PerAF	0	15.8	319	22.0	17.6	529	30
4	68	M	33	PAF	0	11.5	377	29.1	12.5	413	30
5	78	M	30	PAF	0	14.6	376	21.5	13.8	400	27
6	76	M	30	PAF	0	14.0	403	27.0	14.6	537	34
7	79	F	34	PAF	0	15.1	437	28.5	14.5	498	30
8	77	M	38	PAF	2.7	16.8	380	25.6	14	413	25
9	78	M	36	PAF	0	12.2	329	20	12	445	27.5
10	54	M	40	PAF	0	14.2	368	25	10	410	31.2

Abbreviations: AF, atrial fibrillation; LAD, left atrial diameter; LED, lesion‐to‐esophageal distance; PAF, paroxysmal atrial fibrillation; PerAF, persistent atrial fibril1ation; RF radiofrequency.

Table 3 shows the results of the univariate and multivariate analyses of the characteristics of RF lesions associated with PNI. Multivariate logistic analysis revealed that the independent factor of PNI was contact force at an LED of 0–5 mm (odds ratio [OR]: 1.506; 95% confidence interval [CI]: 1.053–2.153; *p* = .025).

**TABLE 3:** Univariate and multivariate logistic regression analysis for predictors of periesophageal vagal nerve injury.

Variables	Univariate		Multivariate	
	Odds ratio (95% CI)	*p* value	Odds ratio (95% CI)	*p* value
Contact force at LED 0–5 mm	1.394 (1.052–1.848)	.021	1.506 (1.053–2.153)	.025
Ablation index at LED 0–5 mm	1.018 (0.997–1.039)	.094	1.010 (0.975–1.046)	.579
Ablation index at LED 5–10 mm	1.013 (0.998–1.028)	.085	0.995 (0.975–1.016)	.632
RF power at LED 0–5 mm	1.284 (0.971–1.698)	.079	1.091 (0.653–1.820)	.740
RF power at LED 5–10 mm	1.353 (0.988–1.853)	.060	1.330 (0.809–2.186)	.261
RF power at LED 10–15 mm	1.238 (0.981–1.563)	.072	1.262 (0.944–1.688)	.116

Abbreviations: CI, confidence interval; HR, hazard ratio; LED, lesion‐to‐esophageal distance; RF, radiofrequency.

**FIGURE 4:**
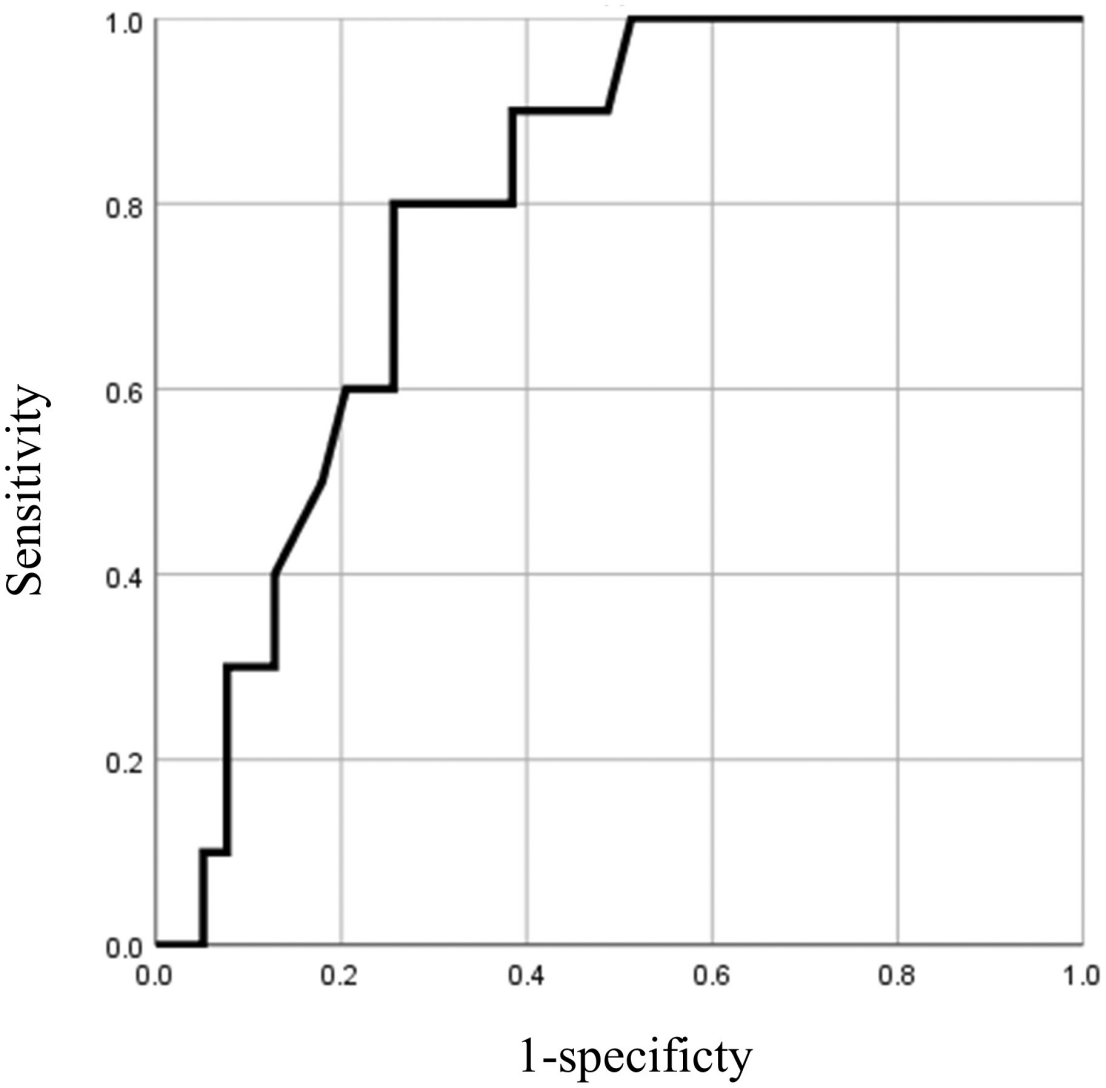
Receiver operating characteristic analysis for contact force at LED 0–5 mm. LED, lesion–esophageal distance.

According to the ROC curve analysis, a contact force at LED 0–5 mm >14 g predicted PNI with a sensitivity of 80% and specificity of 75%, exhibiting an area under the ROC curve of 0.792 (95% CI: 0.663–0.921, *p* < .01) (Figure 4).

### 3.3 Clinical course of patients with periesophageal vagal nerve injury

In patients with PNI, the mean interval from the procedure to symptom onset was 3.2 ± 0.9 days. Eight patients required hospitalization, and the mean duration of hospital treatment was 12.6 ± 6.5 days. Six of eight inpatients received intravenous nutrition for 8.1 ± 4.6 days. Two patients required long‐term parenteral nutrition and underwent the placement of central venous catheters or peripherally inserted central catheters. All the patients recovered completely with conservative treatment. The mean duration of the disappearance of their symptoms was 57.6 ± 75.7 days.

## 4 DISCUSSION

### 4.1 Main findings

In this study, multivariate logistic analysis revealed that the independent factor for symptomatic PNI was the contact force at an LED of 0–5 mm. This finding suggests that the symptomatic PNI is associated with both proximity to the esophagus and a high contact force near the esophagus.

### 4.2 Impact of contact force on periesophageal vagal nerve injury

Radiofrequency applications with high contact forces create deep lesions.^12^, ^13^ Kaneshiro et al.^5^ conducted a comparison of esophageal injury between the high power short duration (HPSD) setting and the conventional power setting of 20 to 30 W. They revealed that the use of the HPSD setting and a mean contact force along the left‐sided posterior isolation line were the independent predictors associated with PNI. A high contact force may cause anatomical distortion of the LA posterior wall, which is in direct contact with the esophagus.^14^ Because the distance between the periesophageal nerves on the surface of the esophagus and the LA posterior wall might become shorter owing to LA distortion, the impact of a high contact force on the periesophageal nerves might be greater. The use of the HPSD setting may mitigate esophageal mucosal injury owing to the shallower RF lesions compared to the conventional power setting; however, it may not be effective in preventing injury to the periesophageal nerves on the surface of the esophagus.

### 4.3 Positional relationship between the esophagus and left atrial

Esophageal injury is related to the proximity of the esophagus to the LA posterior wall.^7^, ^8^, ^15^ The esophagus is usually closest to the LA posterior wall at the level of the left inferior PV orifice.^16^, ^17^, ^18^ The mean distance between the LA posterior wall and bundle of the anterior esophageal plexus was 4.1 ± 1.4 mm.^19^ The distance between the esophagus and the LA posterior wall was shorter in patients with esophageal injury than in those without.^7^ A previous study demonstrated that esophageal injury was associated with a distance below 2.9 mm from the esophagus to the LA posterior wall.^8^ These studies corroborated the finding that RF lesions related to PNI were located within 5 mm of the LED in the present study. We emphasize that this study revealed that the PNI was associated not only with the proximity of RF lesions to the esophagus but also with the characteristics of the RF lesions.

### 4.4 Ablation index and periesophageal vagal nerve injury

Ablation index‐guided AF ablation is instrumental in predicting lesion formation and enhancing the safety of PVI.^20^, ^21^, ^22^ A recent study highlighted an association between the PNI and a higher ablation index value of 380 compared to 320 and 350 at the LA posterior wall.^23^ However, in this study, there was no significant difference observed in the ablation index near the esophagus (LED 0–5 mm) between patients with PNI and without PNI (364 ± 36 vs. 341 ± 36; *p* = .08). The lower ablation index near the esophagus might be attributed to our approach of restricting both RF power and duration to within 30 s or until the esophageal temperature reached >41°C, irrespective of the ablation index. Therefore, we hypothesized that the distortion of LA due to the higher contact force, rather than the creation of larger RF lesions, which was predicted by the ablation index, might have affected the occurrence of PNI.

### 4.5 Esophageal temperature monitoring and periesophageal vagal nerve injury

It has been reported that esophageal temperature monitoring is useful for avoiding esophageal injury^24^; however, we could not prevent PNI. In an ex vivo study, the rise in endoluminal temperature was lower than the rise in epi‐esophageal temperature during RF ablation.^25^ Therefore, the rise in endoluminal temperature might underestimate the rise in epi‐esophageal temperature, which has an impact on the periesophageal vagal nerve located on the epi‐esophageal surface. A previous study also indicated that a significant rise in esophageal temperature was less likely when an esophageal temperature sensor was placed >20 mm away from the RF lesions.^26^ Consequently, while esophageal temperature monitoring is valuable, its ability to predict PNI may not be absolute.

### 4.6 Clinical implications

This study suggests the necessity of avoiding high contact forces near the esophagus to prevent symptomatic PNI. Utilizing 3D images can aid in accurately understanding the positional relationship between the esophagus and the LA posterior wall. Based on the ROC analysis, we recommend limiting the contact force at the LA posterior wall to <14 g, particularly in proximity to the esophagus.

### 4.7 Limitations

This single‐center retrospective study has some limitations that need to be considered. First, our inclusion criteria focused solely on symptomatic patients, potentially overlooking asymptomatic patients with PNI. Second, the accuracy of the 3D electroanatomical mapping image was a limitation of this study. After merging the 3D electroanatomical mapping image with the CT image, we confirmed their alignment using the fluoroscopic image to improve accuracy. Nevertheless, the distance between the RF lesions and the esophagus on the 3D electroanatomical mapping image might be compromised owing to the mobility of the esophagus.^27^, ^28^ The positional relationship between the LA and the esophagus during the procedure might vary compared to when the CT image was performed. We assessed the position of the esophageal temperature probe using the fluoroscopic image and confirmed that the location of the esophagus remained consistent compared to the CT image.

## 5 CONCLUSIONS

Symptomatic PNI is associated with a high contact force at the LA posterior wall in proximity to the esophagus. Strategies for regulating contact force near the esophagus may aid in the prevention of symptomatic PNI.

## CONFLICT OF INTEREST STATEMENT

Authors declare no conflict of interests for this article.

## ETHICS STATEMENT

Ethical approval for this study was obtained from the local Ethics Committee of Gunma Prefectural Cardiovascular Center.

## PATIENT CONSENT STATEMENT

Informed consent was obtained in the opt‐out system.

## REGISTRY AND THE REGISTRATION NUMBER

N/A.

## ANIMAL STUDIES

N/A.

## Supporting information


Table S1.

## Data Availability

The data that support the findings of this study are available from the corresponding author upon reasonable request.
