# Supplementary material for: Characteristics of radiofrequency lesions in patients with symptomatic periesophageal vagal nerve injury after pulmonary vein isolation
Source: J Arrhythm. 2024 Apr 5;40(3):510–7. doi: 10.1002/joa3.13036 (PMC11199810; doi:10.1002/joa3.13036)
Supplement: Supplementary file 1 — Table S1. [file JOA3-40-510-s001.docx]

**Table 1.** Demographic and baseline characteristics between patients with and without periesophageal vagal nerve injury in the entire population.

|  | Patients with PNI  (n = 10) | Patients without PNI  (n = 1381) | P value |
| --- | --- | --- | --- |
| Age, year | 68.6 ± 13.8 | 65.7 ± 9.7 | 0.39 |
| Men, n (%) | 8 (80%) | 981 (77%) | 0.51 |
| Body Mass Index, kg/m^2^ | 19.1 ± 2.5 | 19.9 ± 3.3 | 0.43 |
| Persistent AF, n (%) | 2 (20%) | 681 (48%) | 0.06 |
| Left atrial diameter, mm | 36.3 ± 6.7 | 40.1 ± 1.3 | 0.05 |
| Left Ventricular ejection fraction, (%) | 60.5 ± 8.5 | 60.3 ± 9.9 | 0.96 |
| Esophageal location at the left side of left atrium | 10 (100%) | 1269 (91%) | 0.32 |

Values are presented as n (%), mean ± SD.

AF, atrial fibrillation; PNI, periesophageal vagal nerve injury
